# Excavating Trajectory Planning of a Mining Rope Shovel Based on Material Surface Perception

**DOI:** 10.3390/s23156653

**Published:** 2023-07-25

**Authors:** Yinnan Feng, Juan Wu, Baoguo Lin, Chenhao Guo

**Affiliations:** 1 College of Mechanical and Vehicle Engineering, Taiyuan University of Technology, Taiyuan 030024, China; fengyinnan0052@link.tyut.edu.cn (Y.F.); linbaoguo0043@link.tyut.edu.cn (B.L.); guochenhao0056@link.tyut.edu.cn (C.G.); 2 Shanxi Province Engineer Technology Research Center for Mine Fluid Control, Taiyuan 030024, China; 3 National-Local Joint Engineering Laboratory of Mining Fluid Control, Taiyuan 030024, China

**Keywords:** mining rope shovel (MRS), scaled model, material surface perception, trajectory planning, grey wolf optimizer

## Abstract

The mining rope shovel (MRS) is one of the core pieces of equipment for open-pit mining, and is currently moving towards intelligent and unmanned transformation, replacing traditional manual operations with intelligent mining. Aiming at the demand for online planning of an intelligent shovel excavation trajectory, an MRS excavating trajectory planning method based on material surface perception is proposed here. First, point cloud data of the material stacking surface are obtained through laser radar to perceive the excavation environment and these point cloud data are horizontally calibrated and filtered to reconstruct the surface morphology of the material surface to provide a material surface model for calculation of the mining volume in the subsequent trajectory planning. Second, kinematics and dynamics analysis of the MRS excavation device are carried out using the Product of Exponentials (PoE) and Lagrange equation, providing a theoretical basis for calculating the excavation energy consumption in trajectory planning. Then, the trajectory model of the bucket tooth tip is established by the method of sixth-order polynomial interpolation. The unit mass excavation energy consumption and unit mass excavation time are taken as the objective function, and the motor performance and the geometric size of the MRS are taken as constraints. The grey wolf optimizer is used for iterative optimization to realize efficient and energy-saving excavation trajectory planning of the MRS. Finally, trajectory planning is carried out for material surfaces with four different shapes (typical, convex, concave, and convex–concave). The results of experimental validation show that the actual hoist and crowd forces are essentially consistent with the planned hoist and crowd forces in terms of the peak value and trend variations, verifying the accuracy of the calculation model and confirming that the full bucket rate and various parameters meet the constraints. Therefore, the trajectory planning method based on material surface perception are feasible for application to different excavation conditions.

## 1. Introduction

A mining rope shovel (MRS) is a kind of large mechanical excavator which is widely used in open-cast mining operation; it is one of the core pieces of equipment used in opencast mining [1]. Currently, MRS excavation work is mainly completed through manual operation of four key actions, namely, excavating, rotation, unloading, and returning, which are performed in a loop. However, this method has low digging efficiency, high energy consumption, and a high failure rate, which seriously hinders the application and development of MRS [2,3]. Therefore, there is an urgent need to replace traditional manual operation with intelligent excavation [4] in order to ensure that the MRS can autonomously and efficiently achieve the expected goals in any environment, as well as to establish an intelligent operation mode to ensure efficient, safe, and energy-saving excavation.

The reasonable planning of excavation trajectory is the basis of intelligent MRS. Currently, domestic and foreign scholars have conducted a great deal of research on excavation trajectory planning for excavators. Based on the theory of soil damage, Awuah-Offei et al. [5] constructed a digging resistance model of an electric shovel, analyzed the kinematics and dynamics of the digging process, and optimized the crowd and hoist speed with the unit energy consumption of digging materials as the objective function. Wang X et al. [6,7] proposed a point-to-point (PTP) trajectory planning method with the goal of minimizing the energy consumption per unit volume of mining, and designed an intelligent mining system accordingly. Wei B et al. [8] proposed a new three-degrees-of-freedom parallel excavating mechanism with higher flexibility for the MRS, and optimized the size and excavation trajectory of the new mechanism with the unit excavation energy consumption as the objective function. Bi Q et al. [9] used a multi-target genetic algorithm to optimize the excavation trajectory in stages for electric shovel automation and verified the effectiveness of the optimization method through field tests. Meng Y et al. [10] established a force model of the bucket during the excavation process based on Coulomb theory, optimized the excavation trajectory using the optimal energy consumption, and verified it by EDEM simulation. Zhang T et al. [11] proposed a multi-target trajectory planning framework for an MRS based on a pseudo-spectral method, which was used for autonomous excavation of the MRS and verified by simulation and experiment. Fan J et al. [12,13] proposed a new electro-hydraulic hybrid MRS and optimized its hoist and crowd speeds with the goal of minimizing energy consumption per unit excavation mass. It can be seen from the above research that most domestic and foreign scholars have focused on theoretical analysis for trajectory planning of excavators, and have not combined environmental perception with trajectory optimization. Moreover, the existing research often takes unit excavation energy consumption as the objective function, meaning that the optimization goal is relatively singular.

To address the aforementioned issues, this article takes a 1/30 scale model of the China-made WK series MRS as its research object and proposes an MRS excavating trajectory planning method based on material surface perception. In this method, the point cloud data of the material stacking surface is obtained by lidar in order to perceive the mining environment. The energy consumption per unit mass and the mining time per unit mass are taken as the optimization objectives, and the full bucket rate, speed, and force are taken as constraints. The gray wolf algorithm is used to plan the excavating trajectory. The first part of this paper introduces the research background, the second part analyzes the kinematics and dynamics of the MRS working device, and the third part introduces the method of geometric modeling the material surface using 3D point cloud data. In the fourth part, the trajectory planning model is established and the trajectory planning of the material surface is carried out under various working conditions. In the fifth part, a scale model testbed of the MRS is established and the trajectory planning results are verified by experiments. Finally, the full text is summarized and the paper is concluded.

## 2. Kinematic and Dynamic Analysis

### 2.1. Construction of the MRS Scale Model

**Figure 1:**
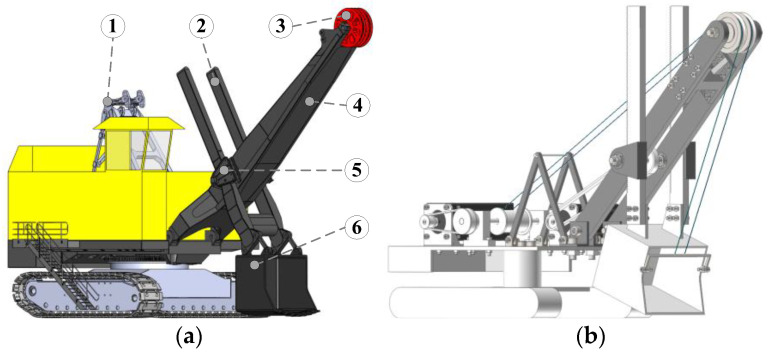
MRS model and MRS scale model: (**a**) MRS model and its main structure (① A frame, ② bucket handle, ③ boom point sheave, ④ boom, ⑤ saddle block, ⑥ bucket); (**b**) MRS scale model.

The study focused on a certain model of WK series MRS made in China. On the basis of the model and based on similarity theory [14,15], an MRS scale model was established at a ratio of 1/30 and an experimental platform for the MRS prototype model was built, as shown in Figure 1. 

**Figure 2:**
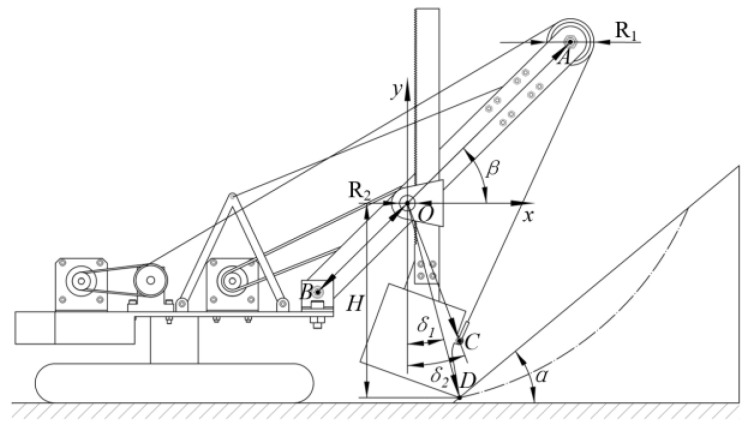
Structural parameters of the MRS scale model [16].

The structural parameters corresponding to the front-end working device of the MRS scale model are defined by the diagram shown in Figure 2 [16]. The specific numerical values of each parameter and the geometric size of the dipper can be found in Table 1 [16].

### 2.2. Kinematic Analysis of the MRS Excavating Device

**Figure 3:**
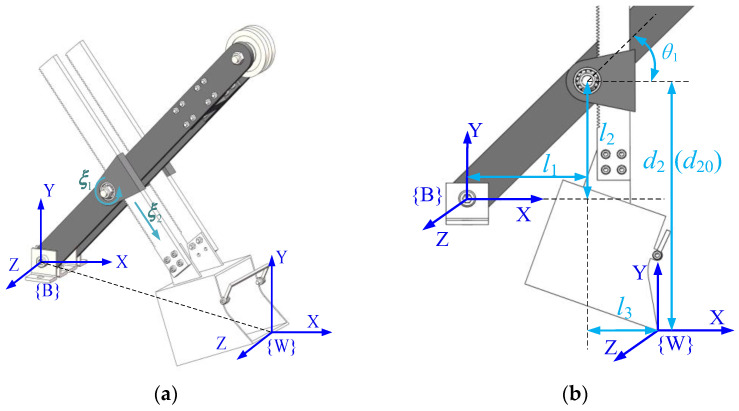
Establishment of coordinate system for the MRS scale model: (**a**) general pose and (**b**) initial pose.

The digging cycle of the MRS consists of four stages: digging, rotating, unloading, and returning. This article mainly carries out trajectory planning for its excavating process. During the excavation process, the upper carriage rotation and the crawler walking mechanism are not working, and the boom remains stationary. Therefore, the MRS can be simplified to a 1R-1P system, that is, the rotary movement of the bucket handle around the $\xi_{1}$ direction and the translational movement along the $\xi_{2}$ direction, as shown in Figure 3a.

Figure 3a,b shows the general pose and initial pose in the process of the MRS excavation, where $l_{1}$ and $l_{2}$ are the horizontal distance and perpendicular distance from the rotary center of saddle block to the coordinate origin, respectively; $l_{3}$ is the distance from the saddle block rotary center to the bucket tooth tip in the perpendicular direction of the bucket handle; $\theta_{1}$ is the rotary angle of the bucket handle; $d_{2}$ is the elongation of the bucket handle; and $d_{20}$ is the initial value of the bucket handle extension length.

In the article, the kinematics modeling and analysis of the working device of the front end of the shovel is carried out using the product of exponentials (PoE) [17,18,19]. The construction of the working device is shown in the above figure. First, the fixed base coordinate system {B} and the end bucket tooth tip coordinate system {W} are established as shown. Second, the following process variables need to be introduced: $T_{BW}(q)$ denotes the pose transformation matrix of {W} relative to {B}; $T_{BW}(0)$ denotes the initial pose transformation matrix of {W} relative to {B}; and $\xi_{1}$ and $\xi_{2}$ represent the joint spinors of the rotating joint and prismatic joint in {B}, respectively. Therefore, the kinematic model from the base coordinate system {B} of the working device to the end bucket tooth tip coordinate system {W} can be expressed as [16].

(1) $$T_{BW}\left(q\right)=e^{\left[{\hat{\xi }}_{1}\right]}{}^{\theta_{1}}e^{\left[{\hat{\xi }}_{2}\right]}{}^{d_{2}}T_{BW}\left(0\right)$$

In the above equation, the expression for the rotational axis spinor is $\xi ={\left(\omega ,\nu \right)}^{T}$, where ν=r×ω, r denotes an arbitrary point on the rotary axis, and ω is the unit vector representing the direction of the rotary axis. The spinor expression for the translational axis is $\xi ={\left(0,\nu \right)}^{T}$, while the kinematic matrix of the rotary axis is [16].

(2) $$\{\begin{array}{c} e^{\left[\hat{\xi }\right]}{}^{\theta }=\left[\begin{array}{cc} e^{\left[\hat{\omega }\right]\theta } & \left(I-e^{\left[\hat{\omega }\right]\theta }\right)\left(\hat{\omega }\times v\right)+\hat{\omega }{\hat{\omega }}^{T}v\theta \\ 0_{1\times 3} & 1 \end{array}\right],\hat{\omega }\neq 0\\ e^{\left[\hat{\xi }\right]}{}^{\theta }=\left[\begin{array}{cc} I & \hat{v}\theta \\ 0 & 1 \end{array}\right],\hat{\omega }=0 \end{array}$$

Therefore, the specific expression of $T_{BW}(q)$ can be obtained by calculation as follows [16]:

(3) $$T_{BW}\left(q\right){=e}^{\left[{\hat{\xi }}_{1}\right]}{}^{\theta_{1}}e^{\left[{\hat{\xi }}_{2}\right]}{}^{d_{2}}T_{BW}\left(0\right)=\left[\begin{array}{cccc} \cos\theta_{1} & -\sin\theta_{1} & 0 & l_{3}\cos\theta_{1}+l_{1}{+d}_{2}\sin\theta_{1}+d_{20}\sin\theta_{1}\\ \sin\theta_{1} & \cos\theta_{1} & 0 & l_{3}\sin\theta_{1}+l_{2}-d_{2}\cos\theta_{1}-d_{20}\cos\theta_{1}\\ 0 & 0 & 1 & 0\\ 0 & 0 & 0 & 1 \end{array}\right]$$

where $l_{1}$, $l_{2}$, and $l_{3}$ are fixed structural parameters. Therefore, by measuring the values of $\theta_{1}$ and $d_{2}$ we can obtain the pose of the bucket tooth tip relative to the body, thereby completing the solution of the forward kinematics problem.

The solution to the inverse kinematics problem for the MRS scaled model is to calculate the joint variables $\theta_{1}$ and $d_{2}$ based on the pose of the bucket tooth tip. Thus, assuming that the bucket pose transform matrix ***T*** is known [16],

(4) $$T=\left[\begin{array}{cccc} a_{11} & a_{12} & a_{13} & b_{x}\\ a_{21} & a_{22} & a_{23} & b_{y}\\ a_{31} & a_{32} & a_{33} & b_{z}\\ 0 & 0 & 0 & 1 \end{array}\right]$$

(5) $$a=[\begin{array}{ccc} a_{11} & a_{12} & a_{13}\\ a_{21} & a_{22} & a_{23}\\ a_{31} & a_{32} & a_{33} \end{array}],\;b=\left[\begin{array}{c} b_{x}\\ b_{y}\\ b_{z} \end{array}\right]$$

In the above formula, *a* is the rotary transformation matrix and *b* is the translational transformation matrix. By comparing (3) and (4), we obtained [16]

(6) $$\begin{array}{l} b_{x}=l_{3}\cos\theta_{1}+l_{1}+d_{2}\sin\theta_{1}+d_{20}\sin\theta_{1}\\ b_{y}=l_{3}\sin\theta_{1}+l_{2}-d_{2}\cos\theta_{1}-d_{20}\cos\theta_{1} \end{array}$$

By solving Equation (6), we can obtain the expressions for $\theta_{1}$ and $d_{2}$ [16]:

(7) $$\theta_{1}=\arctan\left(\frac{\left(b_{y}-l_{2})l_{3}+(b_{x}-l_{1})(d_{2}+d_{20}\right)}{\left(b_{x}-l_{1})l_{3}-(b_{y}-l_{2})(d_{2}+d_{20}\right)}\right)(\theta_{1}\neq 90{}^{\circ})$$

(8) $$d_{2}=\sqrt{{\left(b_{x}-l_{1}\right)}^{2}+{\left(b_{y}-l_{2}\right)}^{2}-l_{3}{}^{2}}-d_{20}$$

Therefore, when the pose transformation matrix from the machine body to bucket tooth tip is known, it is possible to calculate the values of $\theta_{1}$ and $d_{2}$ from Formulas (7) and (8) to solve the inverse kinematics problem.

### 2.3. Dynamic Analysis

**Figure 4:**
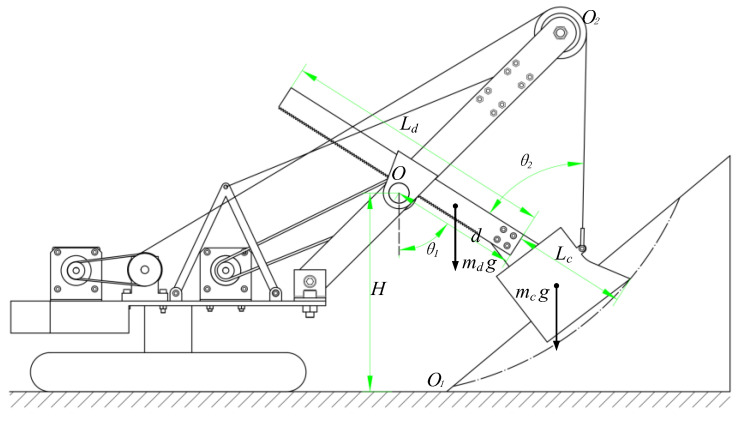
Generalized coordinates of the working device.

As can be seen from the previous kinematics analysis, the working mechanism of the MRS can be simplified to a 1R-1P system, which includes the rotary movement of the bucket handle and bucket around the saddle block rotary center as well as the translational motion along the direction of the bucket handle. Therefore, this article defines the angle $\theta_{1}$ between the bucket handle and the vertical direction and the elongation d of the bucket handle as generalized coordinates, as shown in Figure 4.

In considering the structural characteristics of the working device of the MRS and providing a better calculation model for the subsequent trajectory optimization, this paper adopts the Lagrangian equation to dynamically model the working device of the MRS. The Lagrangian equation of the working device at the front end of the MRS is obtained as follows:

(9) $$\{\begin{array}{c} \frac{d}{dt}\left(\frac{\partial L}{\partial \dot{d}}\right)-\frac{\partial L}{\partial d}=F_{d}\\ \frac{d}{dt}\left(\frac{\partial L}{\partial \dot{\theta }}\right)-\frac{\partial L}{\partial \theta }=F_{\theta } \end{array}$$

In the above equation, L represents the Lagrangian function, which is defined as the difference between the total kinetic energy K and total potential energy P in the relative inertial system, that is, L=K−P, while $F_{d}$ is the resulting force acting on the bucket along the bucket handle direction and $F_{\theta }$ is the resulting force along the tangential direction of the bucket tooth tip. Based on the two generalized coordinates, the dynamic model of the digging device at the front end of the MRS can be obtained as follows:

(10) $$\{\begin{array}{l} \begin{array}{l} \left[m_{d}\left(d^{2}-L_{d}d+\frac{1}{3}L_{d}^{2}\right)+m_{c}\left(d^{2}+L_{c}d+\frac{1}{3}L_{c}^{2}\right)\right]{\ddot{\theta }}_{1}+\left[2d\left(m_{d}+m_{c}\right)-\left(m_{d}L_{d}-m_{c}L_{c}\right)\right]{\dot{\theta }}_{1}\dot{d}\\ +\left[m_{d}g\sin\theta_{1}\left(d-\frac{L_{d}}{2}\right)+m_{c}g\sin\theta_{1}\left(d+\frac{L_{d}}{2}\right)\right]=F_{ti}d\sin\theta_{2}-F_{\tau }\left(L_{c}+d\right) \end{array}\\ \begin{array}{l} \left(m_{d}+m_{c}\right)\ddot{d}-\left[\left(m_{d}+m_{c}\right)d-\frac{1}{2}\left(m_{d}L_{d}-m_{c}L_{c}\right)\right]{\dot{\theta }}_{1}{}^{2}=F_{tui}-F_{n}-F_{ti}\cos\theta_{2}\\ +\left(m_{d}+m_{c}\right)g\cos\theta_{1} \end{array} \end{array}$$

where $m_{d}$ is the mass of the bucket handle; $L_{d}$ is the length of the bucket handle; $m_{c}$ is the mass of the bucket, including the mass of the excavated materials and the weight of the bucket itself; $L_{c}$ is the length of the bucket; $F_{ti}$ is the hoist force of the wire rope; $\theta_{2}$ is the angle between the wire rope and the bucket handle direction; $F_{\tau }$ is the tangential digging resistance; $F_{tui}$ represents the crowd force of the bucket handle; and $F_{n}$ represents the normal digging resistance.

## 3. Material Surface Scanning Based on Laser Radar

To achieve rapid modeling of the material stack surfaces, in this paper we propose a method for using three-dimensional point clouds to rapidly construct a geometric model of the material surfaces. The 3D laser radar emits laser beams towards the material stack and obtains a large amount of point cloud data by calculating the reflection time and propagation speed of the laser. Then, the ordered point cloud is obtained by a combined filtering process to complete the modeling of the material surface.

**Figure 5:**
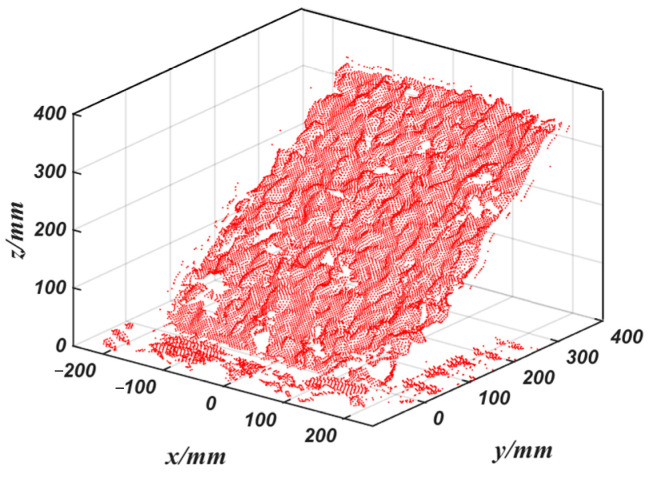
Point cloud data of the material surface.

**Figure 6:**
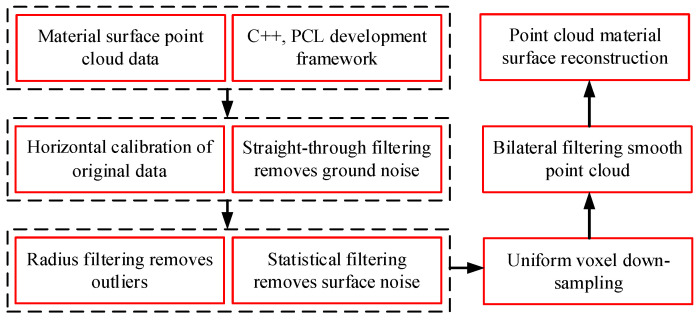
Point cloud processing program.

The original point cloud data of the material stack, as shown in Figure 5, indicate that there is a large amount of noise in the point cloud due to the influence of scanning speed and the surrounding environment, making it difficult to generate an accurate three-dimensional surface model. Therefore, the combined filtering method shown in Figure 6, which was jointly developed based on C++ and the PCL point cloud library, was used to reconstruct the surface.

### 3.1. Horizontal Calibration of Point Cloud Data

Due to the uneven ground of the mine, the point cloud data have an error in the roll angle α and pitch angle β relative to the horizontal plane. Therefore, horizontal calibration was performed before removing the ground points. In the horizontal plane, the Cartesian coordinate system was established perpendicular to the direction of the MRS crawler as the *x*-axis, parallel to the direction of the crawler as the *y*-axis, and vertical to the direction of the horizontal plane as the *z*-axis.

The coordinate transformation matrix is shown below:

(11) $$T=\left(\begin{array}{ccc} \cos\beta & 0 & -\sin\beta \\ \sin\alpha \cdot \sin\beta & \cos\alpha & \sin\alpha \cdot \cos\beta \\ \cos\alpha \cdot \sin\beta & -\sin\alpha & \cos\alpha \cdot \cos\beta \end{array}\right)$$

The formula for correcting the original point cloud data is as follows:

(12) $$\left(\begin{array}{c} x\\ y\\ z \end{array}\right)=T\left(\begin{array}{c} x_{s}\\ y_{s}\\ z_{s} \end{array}\right)$$

In the formula, $x_{s}$, $y_{s}$, and $z_{s}$ are the original point cloud data.

### 3.2. Point Cloud Data Combination Filtering Process

In this paper, the ground noise was filtered by straight-through filtering. Ground points were removed by setting a height threshold. If the z value of a point in the point cloud data was less than the maximum height, it was considered a ground point and discarded. If it was greater than or equal to the maximum height, it was considered a surface point of the material and was retained. This allows for fast removal of outliers, achieving the goal of rough processing in the first step. Additionally, a filter method combining radius and statistical filtering was used to remove surface and outlier noise. The formula used for calculating the Euclidean distance between any two points is as follows:

(13) $$d(p_{i},p_{j})=\sqrt{{\left(x_{i}-x_{j}\right)}^{2}+{\left(y_{i}-y_{j}\right)}^{2}+{\left(z_{i}-z_{j}\right)}^{2}}\leq K_{s}$$

In the equation, x, y, and z represent the three-dimensional coordinates of the point clouds. Radius filtering is used to calculate the Euclidean distance between a point cloud and other point clouds in its spatial neighborhood, and the point cloud is removed or retained by comparing the distance between the two along with the set threshold distance $K_{s}$.

Based on this, the mean Euclidean distance ${\bar{d}}_{i}$ of all point cloud neighborhoods can be calculated according to the number of point clouds in the neighborhood and the global neighborhood mean $\bar{d}$ can be calculated based on the total number of point clouds. Then, the standard deviation of the point cloud neighborhood is calculated as follows:

(14) $$\delta =\sqrt{V}=\sqrt{\frac{1}{n-1}\left(\sum_{i=0}^{n}{\bar{d}}_{i}{}^{2}-\frac{1}{n}{\left(\sum_{i=0}^{n}{\bar{d}}_{i}\right)}^{2}\right)}$$

where V represents the variance of the neighborhood and n represents the total number of point clouds. Assuming that the mean Euclidean distance ${\bar{d}}_{i}$ of the point cloud neighborhood is normally distributed, we have

(15) $${\bar{d}}_{i}\sim N\left(\bar{d},\delta^{2}\right)$$

By setting a filtering threshold factor α, the statistical filtering algorithm removes the point clouds as noise points when the following formula holds:

(16) $$\left\|{\bar{d}}_{i}-\bar{d}\right\|>\alpha \delta$$

After filtering, the number of point cloud data items is on the order of millions, making it difficult to store and process the data. Therefore, the uniform voxel algorithm was used for “down-sampling” of the point cloud data for compression using the following formula to calculate the minimum length of the voxel grid:

(17) $$\{\begin{array}{c} l_{x}=x_{\max}-x_{\min}\\ l_{y}=y_{\max}-y_{\min}\\ l_{z}=z_{\max}-z_{\min} \end{array}$$

where $x_{\max}$, $y_{\max}$, and $z_{\max}$ represent the maximum values of the three-dimensional coordinates of the point cloud and $x_{\min}$, $y_{\min}$, and $z_{\min}$ represent the minimum values. Then, we can use the following formula to calculate the voxel grid size:

(18) $$\{\begin{array}{c} D_{x}=l_{x}/r\\ D_{y}=l_{y}/r\\ D_{z}=l_{z}/r \end{array}$$

where r is the length of the set voxel small grid. The centroid point within the voxel grid is used to replace all points within the grid, achieving the purpose of down-sampling.

After point cloud sampling, the improved bilateral filtering based on normality is used to smooth the surface of the point cloud to eliminate sharp noise caused by individual point cloud fluctuations, improve the quality of the point cloud, and complete surface reconstruction. This algorithm considers the distance between points and neighboring points and uses the distance along the normal direction as a judgment basis. For a certain point P in the point cloud, the unit normal vector of point P is first calculated using the points within the neighborhood range of point P, then the position of P is updated using the following formula:

(19) $$P^{\prime }=P+\delta_{P}\cdot n_{p}$$

where $P^{\prime }$ is the updated position of the point, $n_{P}$ represents the normal vector of point P, and $\delta_{P}$ represents the bilateral filtering factor, as shown in the following equation:

(20) $$\delta_{P}=\frac{\sum {}_{q\in N_{r}{}_{\left(P\right)}}w_{d}\left(\left\|q-P\right\|\right)w_{n}\left(\left|<n_{P},q-P>\right|\right)<n_{P},q-P>}{\sum {}_{q\in N_{r}{}_{\left(P\right)}}w_{d}\left(\left\|q-P\right\|\right)w_{n}\left(\left|<n_{P},q-P>\right|\right)}$$

In the above formula, $N_{r}{}_{\left(P\right)}$ represents the neighborhood of a point P, $w_{d}$ and $w_{n}$ are given Gaussian weights, and q represents the points within the neighborhood P.

**Figure 7:**
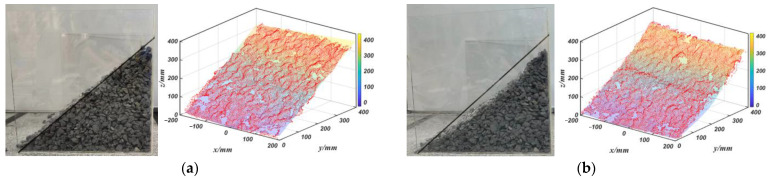

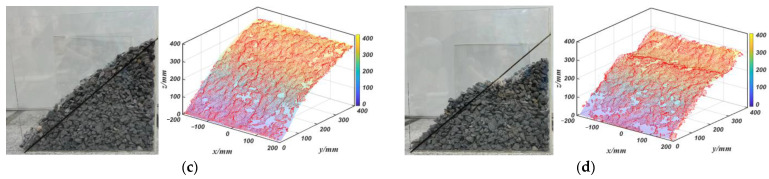
Material stacking surfaces under different working conditions and their surface models: (**a**) typical stacking surface; (**b**) concave stacking surface; (**c**) convex stacking surface; (**d**) convex–concave stacking surface.

Due to the fact that the shape of the material surface is often complex and variable, and may not necessarily be an ideal 40-degree slope [20,21], this paper piled four different shapes of material surfaces and scanned and filtered the material stacking surfaces under different working conditions to obtain the material stacking surfaces model shown in Figure 7. The color bars in the figure represent the vertical height of the material stacking surface from 0–400 mm. The material properties of the material stacking surface are shown in Table 2 below.

To improve the efficiency of surface fitting [22,23], eight points with equal intervals were selected in the X axial direction of the point cloud and another eight points with equal intervals were selected in the Y axial direction. Surface fitting of the material stacking surface was performed using 64 points, with the goodness-of-fit parameter $R^{2}$ used to constrain the fitting effect ($R^{2}$ > 0.95). Finally, the mathematical expression of the material surface in the {Oo} coordinate system was obtained:

(21) z=f(x,y)

## 4. Trajectory Planning Model Based on Grey Wolf Algorithm

Before establishing an optimization model, it is often necessary to determine the three elements of optimization: optimization variables, objective functions, and constraints. In this paper, information on the material stacking surface was obtained through laser scanning and the material surface data were imported into the trajectory planning model. The optimal excavation trajectory under the current operating conditions was obtained by using the grey wolf algorithm to perform optimization calculations.

### 4.1. Determination of Optimization Variables

**Figure 8:**
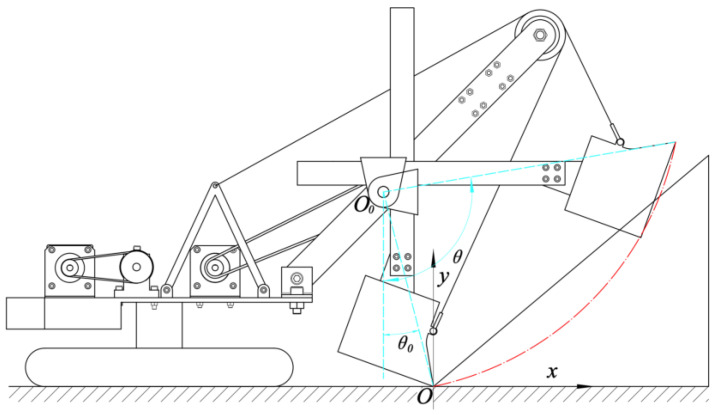
Calculation model of tooth tip excavation trajectory.

In this paper, following the comparative experiments performed by Wang et al. [4], trajectory planning was carried out by using the sixth-order polynomial interpolation method and the excavation process of the MRS was divided into x direction and y direction motion, as shown in Figure 8. The design variables were determined as x = [$a_{x0},a_{x1},a_{x2},a_{x3},a_{x4},a_{x5},a_{x6},a_{y0},a_{y1},a_{y2},a_{y3},$$a_{y4},a_{y5},a_{y6}$] and the excavation trajectory of the MRS can be represented as

(22) $$\{\begin{array}{c} g_{x}\left(t\right)=a_{x6}t^{6}+a_{x5}t^{5}+a_{x4}t^{4}+a_{x3}t^{3}+a_{x2}t^{2}+a_{x1}t+a_{x0}\\ g_{y}\left(t\right)=a_{y6}t^{6}+a_{y5}t^{5}+a_{y4}t^{4}+a_{y3}t^{3}+a_{y2}t^{2}+a_{y1}t+a_{y0} \end{array}$$

The intersection point of the material surface and the horizontal plane, which is the initial position of the bucket tooth tip, was set as the excavation starting point (0,0). Here, the termination position of the tooth tip at the end of the excavation is set to $\left(g_{x},g_{y}\right)$ and the excavation termination time is $t_{d}$. To guarantee the smoothness of the MRS excavating process, the velocities and accelerations of the starting and ending points were both set to 0, that is:

(23) $$\{\begin{array}{c} \begin{array}{c} \begin{array}{c} g_{x}\left(0\right)=0\\ g_{y}\left(0\right)=0 \end{array}\\ v_{x}(0)=0 \end{array}\\ v_{y}(0)=0\\ a_{x}(0)=0\\ a_{y}(0)=0 \end{array}\{\begin{array}{c} \begin{array}{c} \begin{array}{c} g_{x}\left(t_{d}\right)=g_{x}\\ g_{y}\left(t_{d}\right)=g_{y} \end{array}\\ v_{x}(t_{d})=0 \end{array}\\ v_{y}(t_{d})=0\\ a_{x}(t_{d})=0\\ a_{y}(t_{d})=0 \end{array}$$

Therefore, based on the state parameters of the initial and end positions of the bucket tooth tip, the coefficients of the sixth-order polynomial can be processed:

(24) $$\{\begin{array}{c} \begin{array}{c} \begin{array}{c} a_{x0}=0\\ a_{x1}=0 \end{array}\\ a_{x2}=0 \end{array}\\ a_{x3}=\frac{10g_{x}}{t_{d}^{3}}-a_{x6}t_{d}^{3}\\ a_{x4}=-\frac{15g_{x}}{t_{d}^{4}}+3a_{x6}t_{d}^{2}\\ a_{x5}=\frac{6g_{x}}{t_{d}^{5}}-3a_{x6}t_{d} \end{array}\{\begin{array}{c} \begin{array}{c} \begin{array}{c} a_{y0}=0\\ a_{y1}=0 \end{array}\\ a_{y2}=0 \end{array}\\ a_{y3}=\frac{10g_{y}}{t_{d}^{3}}-a_{y6}t_{d}^{3}\\ a_{x4}=-\frac{15g_{y}}{t_{d}^{4}}+3a_{y6}t_{d}^{2}\\ a_{x5}=\frac{6g_{y}}{t_{d}^{5}}-3a_{y6}t_{d} \end{array}$$

Therefore, the sixth-order polynomial only needs to determine the size of $a_{x6},a_{y6},t_{d},g_{x},g_{y}$ to obtain the excavation trajectory of the bucket tooth tip at any time, meaning that the variables to be optimized are x = [$a_{x6},a_{y6},t_{d},g_{x},g_{y}$].

### 4.2. Objective Function Determination

In this paper, the excavation energy consumption and excavation time are comprehensively considered in the excavation trajectory planning and the objective function of trajectory planning is constructed by the unit mass excavation energy consumption and unit mass excavation time of the MRS.

(1) Minimum unit mass excavation energy consumption target

Reducing the excavating energy consumption while meeting the excavating requirements is the main problem of MRS trajectory planning. The energy consumption of the MRS excavating process is mostly the energy consumed by the crowd and hoist motors to overcome the excavation resistance and the gravity of the bucket handle, bucket, and material. Therefore, this paper takes the minimum unit mass excavating energy consumption as the primary objective function of the excavation process; its mathematical expression is

(25) $$f_{1}\left(X\right)=\min\overset{\wedge }{E}=\min\left(\frac{\left[\int {}_{t_{0}}^{t_{d}}v_{h}F_{h}dt+\int {}_{t_{0}}^{t_{d}}v_{c}F_{c}dt\right]}{m_{dig}}\right)$$

Here, $\overset{\wedge }{E}$ represents the total energy consumption during excavation, $v_{h}$ represents the hoist speed, $F_{h}$ represents the hoist force, $v_{c}$ represents the crowd speed of the bucket handle, $F_{c}$ represents the crowd force, and $m_{dig}$ represents the quality of the excavated material.

(2) Maximum excavating efficiency target

To improve the efficiency of MRS excavation, this article takes the time required to excavate the quality of material per unit as another optimization objective. Obviously, the less time it takes to excavate quality material per unit, the higher the excavation efficiency of the MRS. The mathematical expression for this objective is

(26) $$f_{2}(X)=\min(\frac{t_{dig}}{m_{dig}})$$

where $t_{dig}$ is the total excavation time.

(3) Total objective function

To simplify the calculation model used for trajectory planning, a weighted method is adopted to combine the above two objective functions into one. In this way, multi-target optimization is simplified into single-target optimization. According to the role of each objective function in the actual excavating process, the index tolerance method was used to confirm the weight coefficient from the variation range of each objective function, as shown in Table 3. Finally, the weight coefficients of each objective function were set to $k_{1}=0.7$ and $k_{2}=0.3$. The overall objective function is as follows:

(27) $$f(x)=k_{1}f_{1}(X)+k_{2}f_{2}(X)$$

### 4.3. Calculation of Excavation Volume

**Figure 9:**
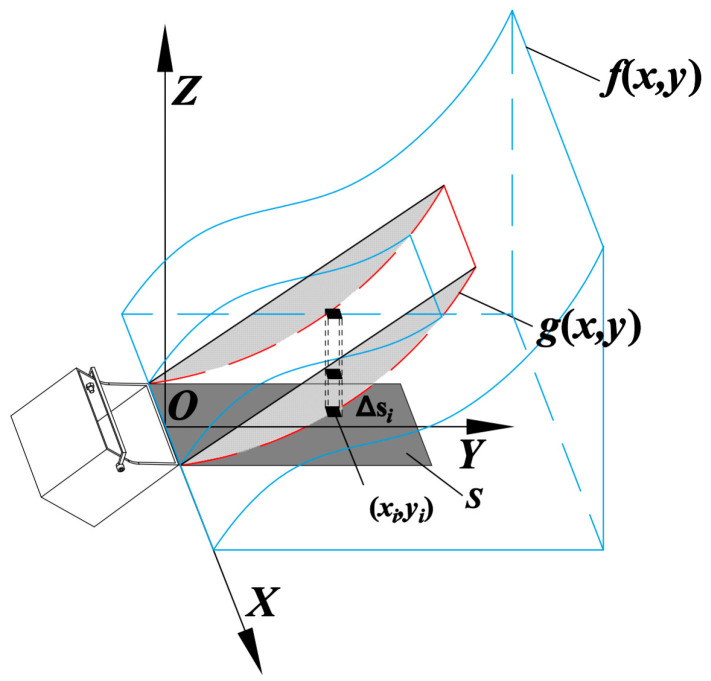
Method for calculating excavation volume.

From the previous processing of point cloud data, we can obtain the expression of the material surface in the {Oo} coordinate system as z=f(x,y). Assuming the optimized excavation surface of the bucket is g(x,y), the excavation volume can be estimated through double integration. The calculation method is shown in Figure 9. The integration domain S is divided into k closed spaces $\Delta s_{i}$; when $\Delta s_{i}$ is very small, a point $\left(x_{i},y_{i}\right)$ can be taken randomly in $\Delta s_{i}$, then the product of $\left[f(x_{i},y_{i})-g(x_{i},y_{i})\right]$ and $\Delta s_{i}$ can be used to calculate the volume of each part. Finally, the volumes of all parts are summed to obtain the excavation volume. The calculation formula is as follows:

(28) $$V=\iint \left[f(x,y)-g(x,y)\right]dxdy=\underset{\max\left|\Delta s\right|\to 0}{\lim}\sum_{i=1}^{k}\left[f(x_{i},y_{i})-g(x_{i},y_{i})\right]\Delta s_{i}$$

### 4.4. Determination of Constraint Conditions

In order to ensure the feasibility of the excavation trajectory, constraint conditions need to be introduced into the MRS trajectory planning model, which can be mainly categorized into excavation performance constraints, motor performance constraints, and geometric dimension constraints, as follows.

(1) Constraint on bucket filling rate

To guarantee the efficiency of the MRS excavating process and to avoid overloading or underloading during excavation, the variation range of the bucket filling rate is limited to 90% to 110%, that is:

(29) $$\left\{ \begin{array}{l} c_{1}=0.9-V/V_{cd}\leq 0\\ c_{2}=V/V_{cd}-1.1\leq 0 \end{array}\right.$$

In the above equation, V represents the volume of the materials excavated by the bucket and $V_{cd}$ is the capacity of the bucket itself.

(2) Constraint on excavation time

To improve the excavating efficiency of the MRS, and based on the relevant literature and actual working conditions, the time range for a single excavation operation of the MRS is set to 9 s to 15 s [11,12], that is:

(30) $$\left\{ \begin{array}{l} c_{3}=9-t_{dig}\leq 0\\ c_{4}=t_{dig}-15\leq 0 \end{array}\right.$$

(3) Digging back angle constraint

To guarantee the stability of the excavation process, the variation range of the digging back angle is limited as follows:

(31) $$\left\{ \begin{array}{l} c_{5}=\beta_{\max}-\beta \leq 0\\ c_{6}=\beta -\beta_{\min}\leq 0 \end{array}\right.$$

where $\beta_{\max}$ and $\beta_{\min}$ represent the upper and lower limits of the digging back angle, respectively.

(4) Velocity constraint

The digging process of the MRS is mainly driven by the crowd and hoist motors. Limited by the performance of the motors, the speed at any time must be less than the maximum output speed of the motor. In the meantime, to ensure the stability of the excavation, the crowd speed and hoist speed at any time should be greater than zero, that is:

(32) $$\left\{ \begin{array}{l} c_{7}=-v_{h}\leq 0\\ c_{8}=v_{h}-v_{h\max}\leq 0\\ c_{9}=-v_{c}\leq 0\\ c_{10}=v_{c}-v_{c\max}\leq 0 \end{array}\right.$$

In the above equation, $v_{h\max}$ and $v_{c\max}$ represent the maximum rotational speeds of the hoist motor and crowd motor.

(5) Driving force and power constraints

To ensure that at any moment during the digging process, neither the crowd and hoist forces nor the crowd and hoist power should exceed the rated value of the motors, that is:

(33) $$\left\{ \begin{array}{l} c_{11}=F_{h}-F_{h\max}\leq 0\\ c_{12}=F_{c}-F_{c\max}\leq 0\\ c_{13}=P_{h}-P_{h\max}\leq 0\\ c_{14}=P_{c}-P_{c\max}\leq 0 \end{array}\right.$$

In the above equation, $F_{h\max}$ and $F_{c\max}$ represent the maximum driving forces of the hoist motor and crowd motor, respectively, while $P_{h\max}$ and $P_{c\max}$ are the respective rated powers of the hoist and crowd motors.

(6) Geometric dimension constraints

The digging cycle of the MRS consists of four stages: digging, rotating, unloading and returning. To guarantee the smooth progress of the subsequent rotating and unloading processes, it is necessary to make sure that the bucket leaves the material surface when the digging is finished and that the bottom of the bucket is higher than the height of the shovel loader hopper, that is,

(34) $$\left\{ \begin{array}{l} c_{15}=H_{dm}-h\leq 0\\ c_{16}=H_{kk}+H_{cd}-h\leq 0 \end{array}\right.$$

where h is the height of the bucket tooth tip, $H_{dm}$ is the height when the bucket tooth tip leaves the material surface, $H_{kk}$ is the height of the shovel loader hopper, and $H_{cd}$ is the height from the bucket tooth tip to the bottom of the bucket.

The maximum stroke of the MRS crowd motion is limited by the size of the bucket handle, while the hoist wire rope is limited by the top sheave of the boom, that is,

(35) $$\left\{ \begin{array}{l} c_{17}=d-L_{dg}\leq 0\\ c_{18}=h-H_{tl}\leq 0 \end{array}\right.$$

where d is the elongation of the bucket handle, $L_{dg}$ is the length dimension of the bucket handle, and $H_{tl}$ is the height of the top sheave of the boom.

Therefore, the optimized design model is as follows:

(36) $$\left\{ \begin{array}{l} x=\left[a_{x6},a_{y6},t_{d},g_{x},g_{y}\right]\\ \min f(x)=k_{1}\left(\frac{\left[\int {}_{t_{0}}^{t_{d}}v_{h}F_{h}dt+\int {}_{t_{0}}^{t_{d}}v_{c}F_{c}dt\right]}{m_{dig}}\right)+k_{2}\frac{t_{dig}}{m_{dig}}\\ c_{i}\leq 0{}^{}(i=1,2,3,\cdots ,18) \end{array}\right.$$

### 4.5. Trajectory Planning Method Based on Grey Wolf Optimizer

The Grey Wolf Optimizer (GWO) is a swarm intelligence optimization algorithm inspired by the social hierarchy and hunting behavior of wolves. It was proposed by Australian scholar Mirjalili et al. [24] in 2014. The algorithm has the advantages of simple structure, few parameters to be adjusted, easy implementation, good solution accuracy and fast convergence speed.

The grey wolf algorithm first constructs the social hierarchy of a wolf pack. The fitness of each individual in the population is calculated and the three wolves with the highest fitness are designated as the α wolf, β wolf, and δ wolf, while the rest of the individuals are labeled as ω wolves. The process of searching for prey (seeking the optimal solution) is mainly achieved through the guidance of the α, β, and δ wolves. During the iterative calculation process, the three wolves α β δ with the highest fitness in the current population are preserved and the positions of other candidate wolves ω are continuously adjusted based on the coordinates of these three wolves; hunting (optimization) is completed by searching for prey, encircling prey, and attacking prey, and finally a set of optimal solutions is obtained. The specific mathematical model is shown below:

(37) $$\begin{array}{l} {\vec{D}}_{\alpha }=\left|{\vec{C}}_{1}\cdot {\vec{X}}_{\alpha }-\vec{X}\right|,{\vec{D}}_{\beta }=\left|{\vec{C}}_{2}\cdot {\vec{X}}_{\beta }-\vec{X}\right|,{\vec{D}}_{\delta }=\left|{\vec{C}}_{3}\cdot {\vec{X}}_{\delta }-\vec{X}\right|\\ {\vec{X}}_{1}={\vec{X}}_{\alpha }-{\vec{A}}_{1}\cdot {\vec{D}}_{\alpha },{\vec{X}}_{2}={\vec{X}}_{\beta }-{\vec{A}}_{2}\cdot {\vec{D}}_{\beta },{\vec{X}}_{3}={\vec{X}}_{\delta }-{\vec{A}}_{3}\cdot {\vec{D}}_{\delta }\\ \vec{X}\left(t+1\right)=\frac{{\vec{X}}_{1}+{\vec{X}}_{2}+{\vec{X}}_{3}}{3} \end{array}$$

**Figure 10:**
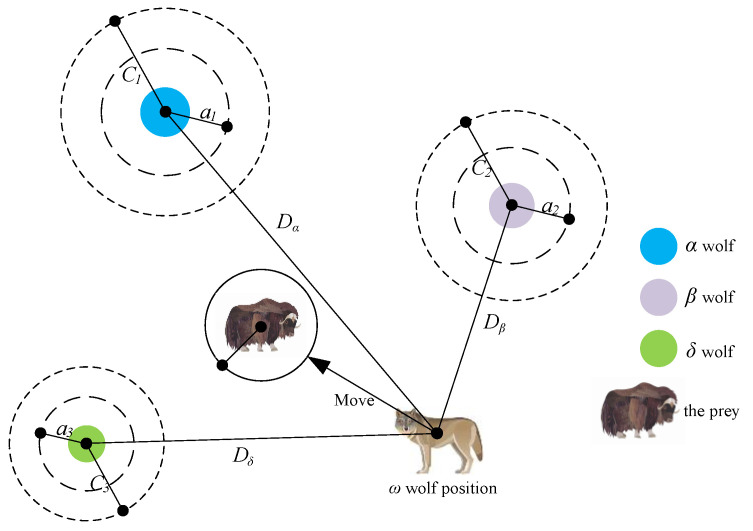
Position updating strategy of GWO.

Here, ${\vec{D}}_{\alpha }$, ${\vec{D}}_{\beta }$ and ${\vec{D}}_{\delta }$ are the distance between the current candidate wolf and the optimal solution (α,β,δ wolves); ${\vec{X}}_{\alpha }$, ${\vec{X}}_{\beta }$, and ${\vec{X}}_{\delta }$ denote the position vectors of the α,β,δ wolves in the current grey wolf population; $\vec{X}$ denotes the position vector of the candidate wolf; t is the current iteration number; $\vec{A}$ and $\vec{C}$ are cooperativity coefficient vectors. Figure 10 illustrates the position updating strategy of GWO in a two-dimensional space.

**Figure 11:**
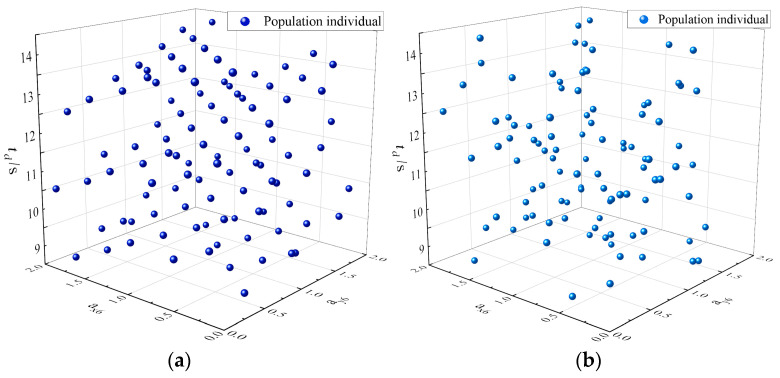
Different initial population generation methods: (**a**) the good point set method and (**b**) the random method.

**Figure 12:**
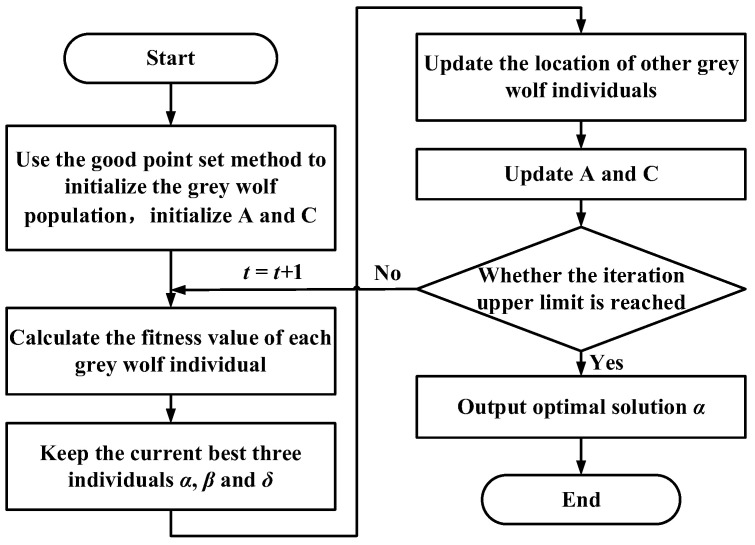
Grey wolf algorithm trajectory planning process.

Because the trajectory planning is for the intelligent operation mode service, it needs to meet real-time requirements; thus, the initialization of the population is particularly important. This article adopts the good point set method to obtain uniformly distributed initial values and then calculates iteratively using the GWO, which improves the convergence speed of the algorithm and can avoid its falling into local optima. Figure 11 shows the grey wolf population generated by the good point set method and by the random method in a three-dimensional search space. It can be seen from the figure that the initial population generated by the random method cannot be uniformly distributed in the entire search space and has strong randomness, making it easy for the algorithm to fall into local optima. In contrast, the initial population generated by the good point set method can be uniformly distributed in the entire search space, enriching the population diversity and enhancing the global search capability of the algorithm. The improved GWO process is shown in Figure 12.

### 4.6. Results of Trajectory Planning

**Figure 13:**
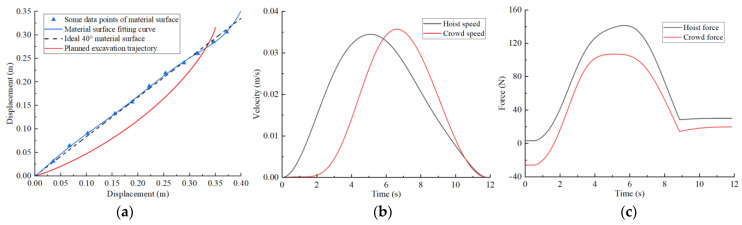
Typical material surface trajectory planning results: (**a**) excavation trajectory; (**b**) hoist and crowd speeds; (**c**) hoist and crowd forces.

**Figure 14:**
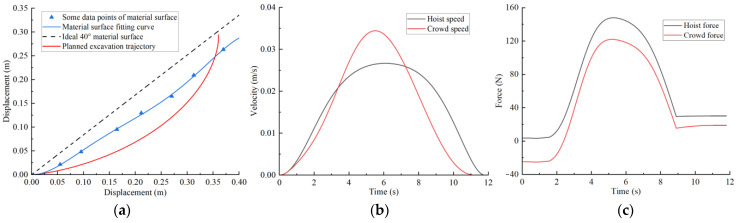
Concave material surface trajectory planning results: (**a**) excavation trajectory; (**b**) hoist and crowd speeds; (**c**) hoist and crowd forces.

**Figure 15:**
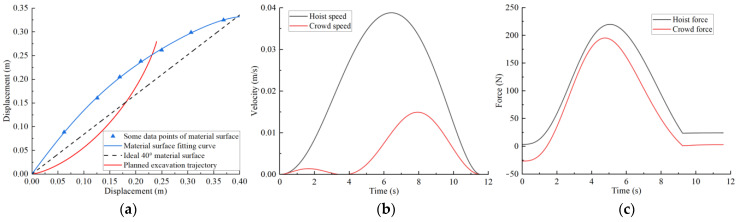
Convex material surface trajectory planning results: (**a**) excavation trajectory; (**b**) hoist and crowd speeds; (**c**) hoist and crowd forces.

**Figure 16:**
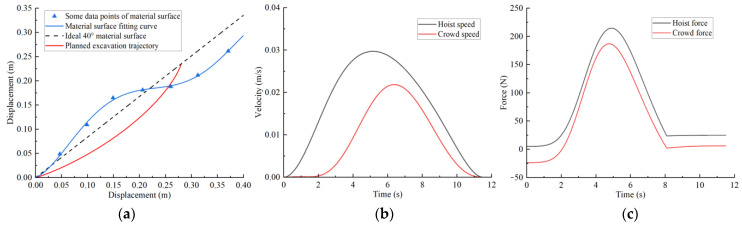
Convex–concave material surface trajectory planning results: (**a**) excavation trajectory; (**b**) hoist and crowd speeds; (**c**) hoist and crowd forces.

Combining the improved grey wolf algorithm process, the basic parameters are set as follows: initial wolf population size pop = 50, search coefficient c = 2, and maximum iterations t_max_ = 100. After trajectory planning, the trajectory planning results for different excavation conditions are shown in Figure 13, Figure 14, Figure 15 and Figure 16. The planned results indicate that the trajectory planning method based on the grey wolf algorithm can be applied to different excavation conditions.

Figure 13a shows the optimal excavating trajectory under different excavation conditions; the abscissa represents the displacement in the horizontal direction, the ordinate represents the displacement in the vertical direction, the blue line represents the material surface fitting curve, the red line represents the planned excavation trajectory, and the black line represents the ideal 40° material surface. Figure 13b shows the crowd speed and hoist speed for various material surface. Figure 13c shows the crowd force and hoist force for various material surface. Figure 13 displays the planning results for a typical material surface, while Figure 14 shows the planning result for a concave material surface. Compared with the typical material surface, with the concave surface the hoist speed is significantly reduced and the excavation trajectory is deeper. Figure 15 shows the planning results for a convex material surface. Compared with the typical material surface, the crowd speed decreases, the hoist speed increases, and the excavation trajectory is shallower. Finally, Figure 16 shows the planning results for a convex–concave material surface. Compared with the typical material surface, the crowd and hoist speed are reduced and the excavation trajectory is between that of the typical material surface and the convex material surface.

Table 4 and Table 5 show the optimization results obtained from trajectory planning under different excavating conditions. It can be found that the shallower the excavation trajectory, the less excavation time required; in addition, the convex–concave material surface requires the least excavating time and energy consumption. All optimization variables satisfy the constraint conditions, and the bucket fill factor meets the requirements; therefore, the trajectory planning method based on the grey wolf algorithm is applicable to different excavating conditions and has certain feasibility and reliability.

## 5. Experimental Verification

**Figure 17:**
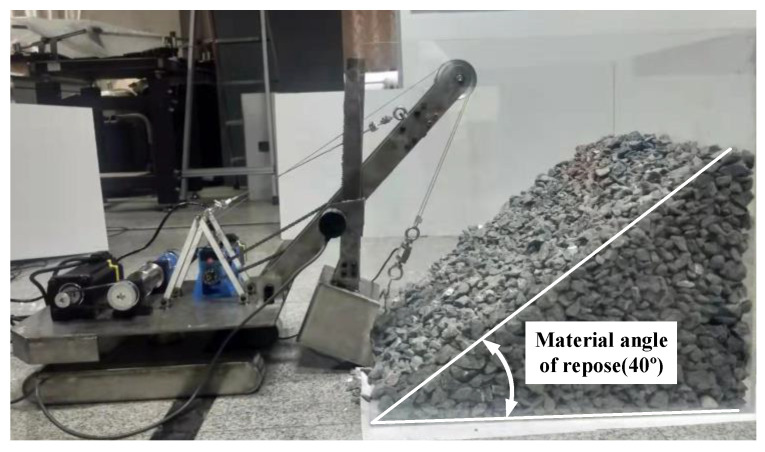
The scaled model testbed of the MRS.

In this study, a scale model testbed of the MRS (as shown in Figure 17) was constructed for excavation testing. The feasibility and credibility of the trajectory planning method were then validated by comparing the planned and tested results.

**Figure 18:**
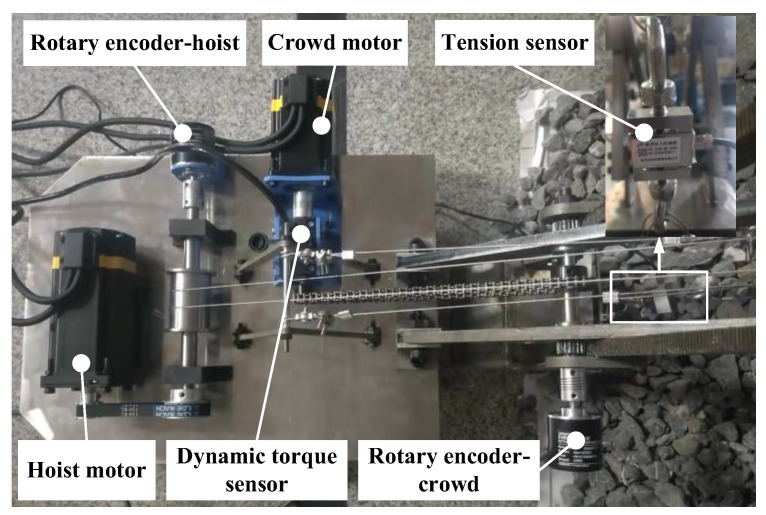
Installation positions of the motors and sensors.

**Figure 19:**
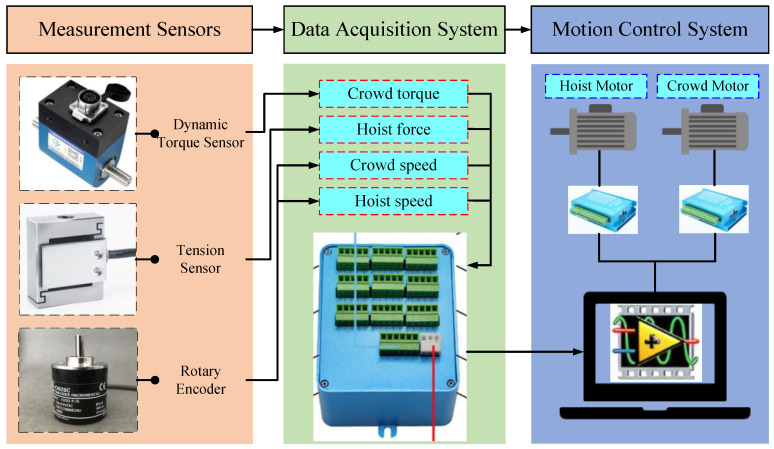
System structure of the MRS scale model testbed.

The hoist and crowd movements of the digging device in the prototype were achieved by individual motor driven systems. The motor control was implemented through an upper computer, LabVIEW 2020 software, and a microcontroller. Dynamic torque sensors were employed to measure the real-time crowd force of the bucket handle, tension sensors to measure the real-time hoist force of the wire rope, and rotation encoders to measure the extension of the bucket handle and the lifting distance of the wire rope. LabVIEW software in cooperates with a data collector was used to realize the acquisition, storage, and display of sensor measurement data. The installation position of the motors and sensors is shown in Figure 18. The structure of the testbed system is shown in Figure 19.

To guarantee the credibility and stability of the test results, it is necessary to repeatedly take the average of trajectory planning tests under different excavating conditions. The data obtained from the torque and tensile sensors were processed using Matlab R2018a, resulting in the curves of crowd force and hoist force over time.

**Figure 20:**
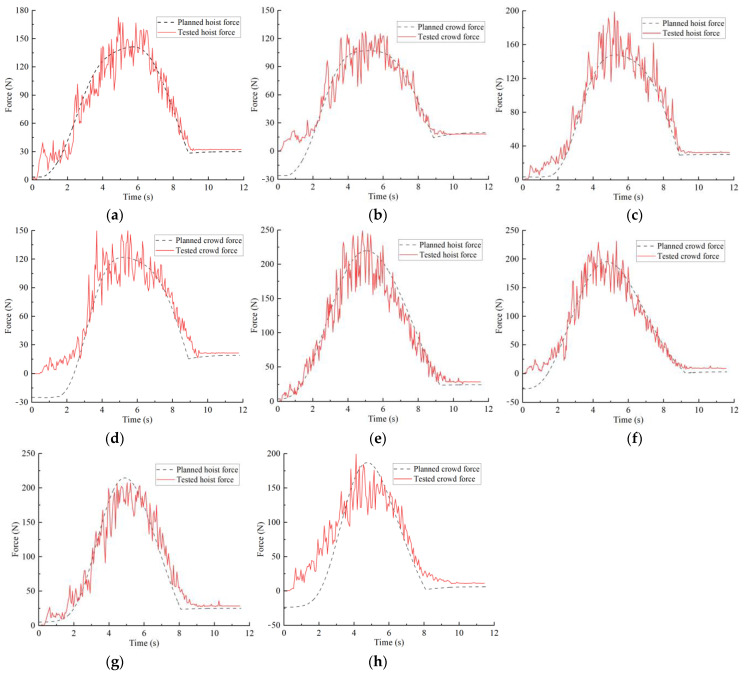
Comparison of different material surface shape planning and test crowd and hoist forces: (**a**) typical material surface hoist force; (**b**) typical material surface crowd force; (**c**) concave material surface hoist force; (**d**) concave material surface crowd force; (**e**) convex material surface hoist force; (**f**) convex material surface crowd force; (**g**) convex–concave material surface hoist force; (**h**) convex–concave material surface crowd force.

Figure 20 compares the planned results and test results of the crowd and hoist forces under different excavation conditions. Due to the flexibility of the hoist wire rope and the resistance fluctuations during the excavation process, the test results are presented as an obviously fluctuating curve. Compared with the planned results, it can be seen that the values of the two are not much different and that the overall change trend is essentially the same. At the same time, the excavating quality and excavating time of the planned and test were compared to validate the credibility and feasibility of the trajectory planning method. Relevant coefficients ($R^{2}$) were introduced to describe the degree of match between the planned and tested results, as shown in Table 6.

As shown in the table, the relative deviations between the planned and tested excavation quality under different excavation conditions are about 5%. The relative deviations of the digging times are below 1%. Additionally, the $R^{2}$ values for the planned and tested results of the crowd force and the hoist force are greater than 0.85, indicating that the two have a high degree of agreement. The test results show that the trajectory planning method based on material stack surface perception has certain feasibility and reliability, that the established dynamic model can more accurately predict the crowd and hoist force, and that the planned results of various performance indicators are reliable.

## 6. Conclusions

(1) A laser radar was used to obtain the point cloud data of the material stack surface in order to perceive the excavating environment, and the point cloud data were horizontally calibrated and filtered to establish a prediction model of the material stack surface. Furthermore, kinematic and dynamic analyses of the MRS excavation device were conducted using the Product of Exponentials and Lagrange equation.
(2) A trajectory planning method for the MRS excavation based on material surface perception and the Grey Wolf Algorithm was proposed, with the unit mass excavation energy consumption and unit mass excavation time as the target functions and the electric motor performance and MRS geometry size as constraints. Trajectory planning was conducted on four different shapes (typical, concave, convex, and convex–concave) of material stack surfaces.
(3) An MRS scale model testbed was constructed and used for experimental verification. The test results show that the planned results for hoist force and crowd force were generally consistent with the test results in terms of the values and change trend under different excavation conditions and had $R^{2}$ values greater than 0.85, validating the feasibility and reliability of the proposed trajectory planning method.

This method can provide a theoretical basis for the intelligent and unmanned development of excavating machinery such as electric shovels and excavators, and has certain feasibility and applicability. In follow-up research, the initial pose of the MRS could be incorporated into the trajectory planning scope. Meanwhile, the point cloud processing and trajectory planning algorithm should be further improved and optimized in order to reduce the optimization time and improve computational efficiency to better meet practical needs.

## Figures and Tables

**Table 1:** Structural parameter values [16].

Parameter (Unit)	Value	Parameter (Unit)	Value
*R*_1_ (mm)	58	*H* (mm)	247.3
*R*_2_ (mm)	20	*α* (°)	40
*l_AB_* (mm)	450	*β* (°)	45
*l_OB_* (mm)	160	*δ*_1_ (°)	14.8
*l_OC_* (mm)	186.5	*δ*_2_ (°)	20.43
*l_OD_* (mm)	255.8	Dipper (mm)	120 × 110 × 100

**Table 2:** Material properties of the material stacking surface.

Material	Poisson’s Ratio	Shear Modulus (MPa)	Density (kg/m^3^)
Limestone	0.35	1.35 × 10^3^	2540

**Table 3:** The weighting coefficients of each objective function.

Objective Function	Variation Range	Tolerance	Weight Coefficient	Normalization
$f_{1}$	[13, 15]	1	1.0000	0.6960
$f_{2}$	[3.75, 8.33]	2.29	0.4367	0.3040

**Table 4:** Different material surface optimization variables.

Different Material Surface	*a_x_*_6_ (10^−7^)	*a_y_*_6_ (10^−7^)	*g_x_* (m)	*g_y_* (m)	*t_d_* (s)
Typical material surface	−9.98	5.41	0.3489	0.3159	11.85
Concave material surface	−17.05	9.73	0.3605	0.2943	11.81
Convex material surface	−5.52	11.33	0.2413	0.2796	11.57
Convex-concave material surface	−9.71	4.15	0.2825	0.2352	11.49

**Table 5:** Different material surface optimization results.

Different Material Surface	*f*	*f*_1_ (J/kg)	*f*_2_ (s/kg)	*m*_dig_ (kg)	Bucket Fill Factor (%)
Typical material surface	13.76	17.19	5.771	2.053	99.72
Concave material surface	13.83	17.22	5.925	1.991	96.71
Convex material surface	13.87	17.51	5.367	2.156	104.7
Convex-concave material surface	12.57	15.55	5.625	2.045	99.29

**Table 6:** Comparison between planned results and experimental results for various excavation conditions.

Different Material Surface	Excavating Quality/kg		Relative Deviation/%	Digging Time/s		Relative Deviation/%	Relevant Coefficient (*R*^2^)	
	Planned Results	Test Results		Planned Results	Test Results		Hoist Force	Crowd Force
Typical material surface	2.053	2.086	1.58	11.85	11.81	0.34	0.9224	0.8861
Concave material surface	1.991	2.079	4.23	11.81	11.72	0.77	0.9103	0.8717
Convex material surface	2.156	2.245	3.96	11.57	11.49	0.70	0.9271	0.9049
Convex-concave material surface	2.045	2.174	5.93	11.49	11.43	0.52	0.9347	0.8531

## Data Availability

Not applicable.

## References

[1] Bi Q., Wang G., Yang R., Liu Y., Lu Y., Xing S. (2019). Study on theory and methods of payload online estimation for cable shovels. J. Braz. Soc. Mech. Sci. Eng..

[2] Babaei Khorzoughi M., Hall R. (2016). A study of digging productivity of an electric rope shovel for different operators. Minerals.

[3] Papic L., Kovacevic S. Human factor in mining machines maintenance operations. Proceedings of the 2016 Second International Symposium on Stochastic Models in Reliability Engineering, Life Science and Operations Management (SMRLO).

[4] Zhang T., Fu T., Cui Y., Song X. (2022). Toward autonomous mining: Design and development of an unmanned electric shovel via point cloud-based optimal trajectory planning. Front. Mech. Eng..

[5] Awuah-Offei K., Frimpong S. (2007). Cable shovel digging optimization for energy efficiency. Mech. Mach. Theory.

[6] Wang X., Sun W., Li E., Song X. (2018). Energy-minimum optimization of the intelligent excavating process for large cable shovel through trajectory planning. Struct. Multidiscip. Optim..

[7] Wang X., Song X., Sun W. (2021). Surrogate based trajectory planning method for an unmanned electric shovel. Mech. Mach. Theory.

[8] Wei B., Gao F. Digging Trajectory Optimization for a New Excavating Mechanism of Electric Mining Shovel. Proceedings of the International Design Engineering Technical Conferences and Computers and Information in Engineering Conference.

[9] Bi Q., Wang G., Wang Y., Yao Z. (2020). Digging Trajectory Optimization for Cable Shovel Robotic Excavation based on a Multi-Objective Genetic Algorithm. Energies.

[10] Meng Y., Fang H., Liang G., Gu Q., Liu L. (2019). Bucket Trajectory Optimization under the Automatic Scooping of LHD. Energies.

[11] Zhang T., Fu T., Song X., Qu F. (2022). Multi-objective excavation trajectory optimization for unmanned electric shovels based on pseudospectral method. Autom. Constr..

[12] Fan R., Li Y., Yang L. Trajectory Planning Based on Minimum Input Energy for the Electro-Hydraulic Cable Shovel. Proceedings of the 2020 IEEE/ASME International Conference on Advanced Intelligent Mechatronics (AIM).

[13] Fan R., Li Y., Yang L. (2022). Multiobjective trajectory optimization of intelligent electro-hydraulic shovel. Front. Mech. Eng..

[14] Coutinho C.P., Baptista A.J., Rodrigues J.D. (2016). Reduced scale models based on similitude theory: A review up to 2015. Eng. Struct..

[15] Ramu M., Prabhu Raja V., Thyla P.R. (2013). Establishment of structural similitude for elastic models and validation of scaling laws. KSCE J. Civ. Eng..

[16] Feng Y., Wu J., Guo C., Lin B. (2022). Numerical Simulation and Experiment on Excavating Resistance of an Electric Cable Shovel Based on EDEM-RecurDyn Bidirectional Coupling. Machines.

[17] Wang H., Lu X., Sheng C., Zhang Z., Cui W., Li Y. (2018). General frame for arbitrary 3R subproblems based on the POE model. Robot. Auton. Syst..

[18] He R., Zhao Y., Yang S. (2010). Kinematic-Parameter Identification for Serial-Robot Calibration Based on POE Formula. IEEE Trans. Robot..

[19] Chen G., Wang H., Lin Z. (2014). Determination of the Identifiable Parameters in Robot Calibration Based on the POE Formula. IEEE Trans. Robot..

[20] Wu J., Wang G., Bi Q., Hall R. (2020). Digging force and power consumption during robotic excavation of cable shovel: Experimental study and DEM simulation. Int. J. Min. Reclam. Environ..

[21] Song X., Zhang T., Yuan Y., Wang X., Sun W. (2020). Multidisciplinary co-design optimization of the structure and control systems for large cable shovel considering cross-disciplinary interaction. Proc. Inst. Mech. Eng. Part C J. Mech. Eng. Sci..

[22] Zhou J., Jin W., Wang M., Liu X., Li Z., Liu Z. (2023). Improvement of normal estimation for point clouds via simplifying surface fitting. Comput.-Aided Des..

[23] Sun J., Xiang S., Zhou T., Cheng T. (2023). Sampling Point Planning for Complex Surface Inspection based on Feature Points under Area Division. Int. J. Adv. Manuf. Technol..

[24] Mirjalili S., Mirjalili S.M., Lewis A. (2014). Grey wolf optimizer. Adv. Eng. Softw..

